# Artificial intelligence (AI) in biomedical research: discussion on authors’ declaration of AI in their articles title

**DOI:** 10.1186/s41747-022-00316-7

**Published:** 2023-01-16

**Authors:** Francesco Sardanelli, Isabella Castiglioni, Anna Colarieti, Simone Schiaffino, Giovanni Di Leo

**Affiliations:** 1 Department of Biomedical Sciences for Health, Università degli Studi di Milano, Via Luigi Mangiagalli 31, 20133 Milan, Italy; 2 Unit of Radiology, IRCCS Policlinico San Donato, San Donato Milanese, Milan, Italy; 3 Department of Physics, Università degli Studi di Milano-Bicocca, Piazza della Scienza 3, 20126 Milan, Italy; 4 Institute of Biomedical Imaging and Physiology, Consiglio Nazionale delle Ricerche, Via Fratelli Cervi 93, 20090 Segrate, Italy

**Keywords:** Artificial intelligence, Big data, Deep learning, Machine learning, Regression analysis

## Abstract

Artificial intelligence (AI) and its different approaches, from machine learning to deep learning, are not new. We discuss here about the declaration of AI in the title of those articles dealing with AI. From 1990 to 2021, while AI articles in the PubMed increased from 300 to 59,596, the percentage declaring AI in the title describes a U-like-shaped curve: about 30% in early 1990s, less than 13% in 2005–2014, again 30% in 2020–2021. A similar trend was observed for AI in medical imaging. While the initial decline could be due to the establishment of AI methods, the recent increase could be related to the capacity of AI to outperform humans, especially in image recognition, fuelled by the adoption of graphic processing units for general purpose computing. The recent increase may also be due to the relevance of open issues about AI, including the standardisation of methods, explainability of results, and concerns about AI-induced epoch-making transformations: to say “We are using AI” in the title may also reflect these concerns.

## Key points

- The recent availability of big data and high computing power allowed an exponential growth of artificial intelligence (AI) research and its application to biomedicine and radiology.
- AI declaration in the article titles dropped from about 30% in early 1990s to less than 13% in 2005–2014 and reached again 30% in 2020–2021.
- While the initial decline could be due to the establishment of AI methods, the recent increase could be due to both expectations and concerns about AI-induced epochal changes.

## Introduction

Artificial intelligence (AI), in all its current approaches, such as machine learning and deep learning [1, 2], has become one of the most important topics for researchers that are looking for innovative ways to leverage data assets to a new level of understanding and usage. The term “AI” was introduced more than 60 years ago, when John McCarthy organised a seminar in 1956 about automata theory, neural networks, and the study of intelligence [3]. One of the participants was the IBM researcher Arthur Lee Samuels, who subsequently developed a self-learning algorithm for playing checkers and in 1959 coined the term *machine learning* [4].

Technological advances in computer vision, speech recognition, and robotics fuelled the introduction of AI in several fields. An increasing concern regards the perspective that AI may displace human workers and make tight surveillance techniques easier to develop. Ethan Fast and Eric Horvitz have presented an understanding of the public hopes and concerns by screening 30 years of publications of the *New York Times* (from January 1986 to June 2016) [5]. Authors showed that AI has had consistently more optimistic than pessimistic coverage over time, especially in healthcare. In addition, the fear of loss of control of AI, for example, has become far more common in recent years.

AI applications were made in the early 1980s for gaming and later for e-commerce, spam email filtering, economics, and biochemistry. In healthcare and biomedicine, AI applications were proposed in the study of different diseases and imaging technologies [6–9], although up until 2000 they were not so frequently referred to as *AI* [10]. Indeed, we randomly noticed articles in the biomedical field that used AI techniques but not declaring it in the title, so that they could not be recognised as being AI articles at first glance.

As the declaration of the method in an article title is somewhat labelling a study—as usually happens for “randomised controlled trials”—we investigated about this particular issue: *the declaration of AI in the title of articles using AI in biomedicine*. This can stimulate some reflections about the role and perception of AI in biomedical research and correlates to the question of the relevance of an article method *versus* the relevance of its results. In other words, can we consider AI as *labelling* a publication or is the used AI technique only a method to reach the study aims?

### Declaring AI in the article title

**Fig. 1:**
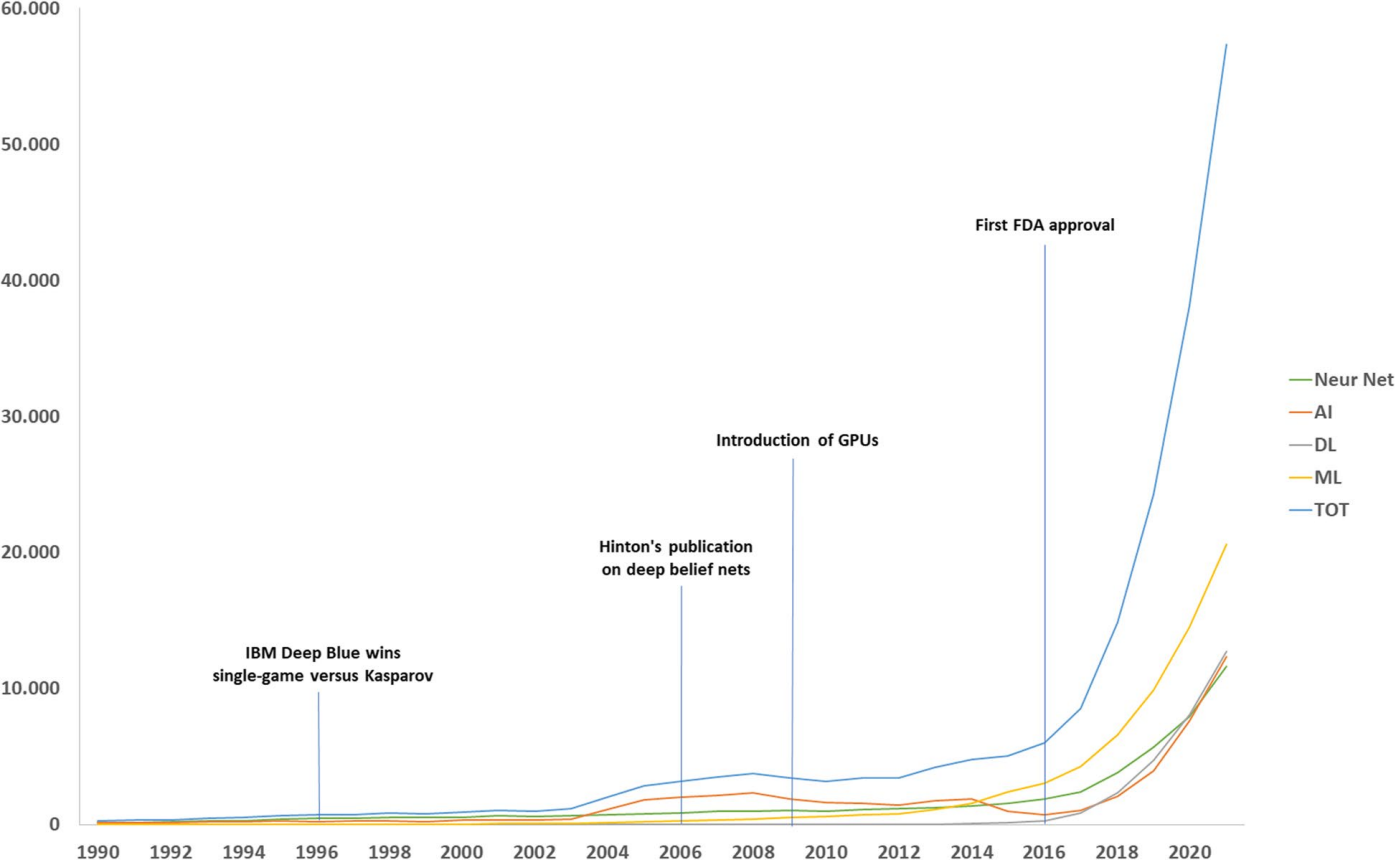
Number of articles dealing with artificial intelligence, overall and by technique. Four major milestones are shown. *Neur Net* Neural network, *AI* Artificial intelligence, *DL* Deep learning, *FDA* Food and Drug Administration, *GPU* Graphics processing units, *ML* Machine learning, *TOT* Total

**Fig. 2:**
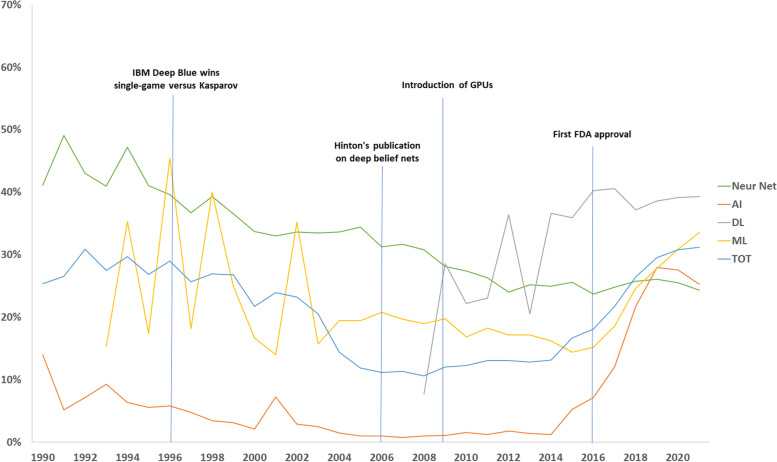
Rate of articles dealing with artificial intelligence and declaring it in the title, overall and by technique. Four major milestones are shown. *Neur Net* Neural network, *AI* Artificial intelligence, *DL* Deep learning, *FDA* Food and Drug Administration, *GPU* Graphics processing unit, *ML* Machine learning, *TOT* Total

On October 20, 2022, we searched for articles using AI methods available in PubMed from January 1990 to December 30, 2021, using the string (“artificial intelligence” OR “machine learning” OR “deep learning” OR “neural network”) (Fig. 1). Then, we calculated the percentage of them declaring AI approaches in the title (Fig. 2).

The absolute number of articles clearly increased exponentially in the last decades. This is a relevant trend but also an effect of the increase in the whole world research activity, regardless of the topic. Interestingly, the total percentage curve appears to have a U-like shape, with a decrease from about 30% in 1990s to 11% in 2008, followed by a steady state up to 2014, and a faster increase until 2020–2021, when the curve seems to be reaching a peak of about 31%.

**Fig. 3:**
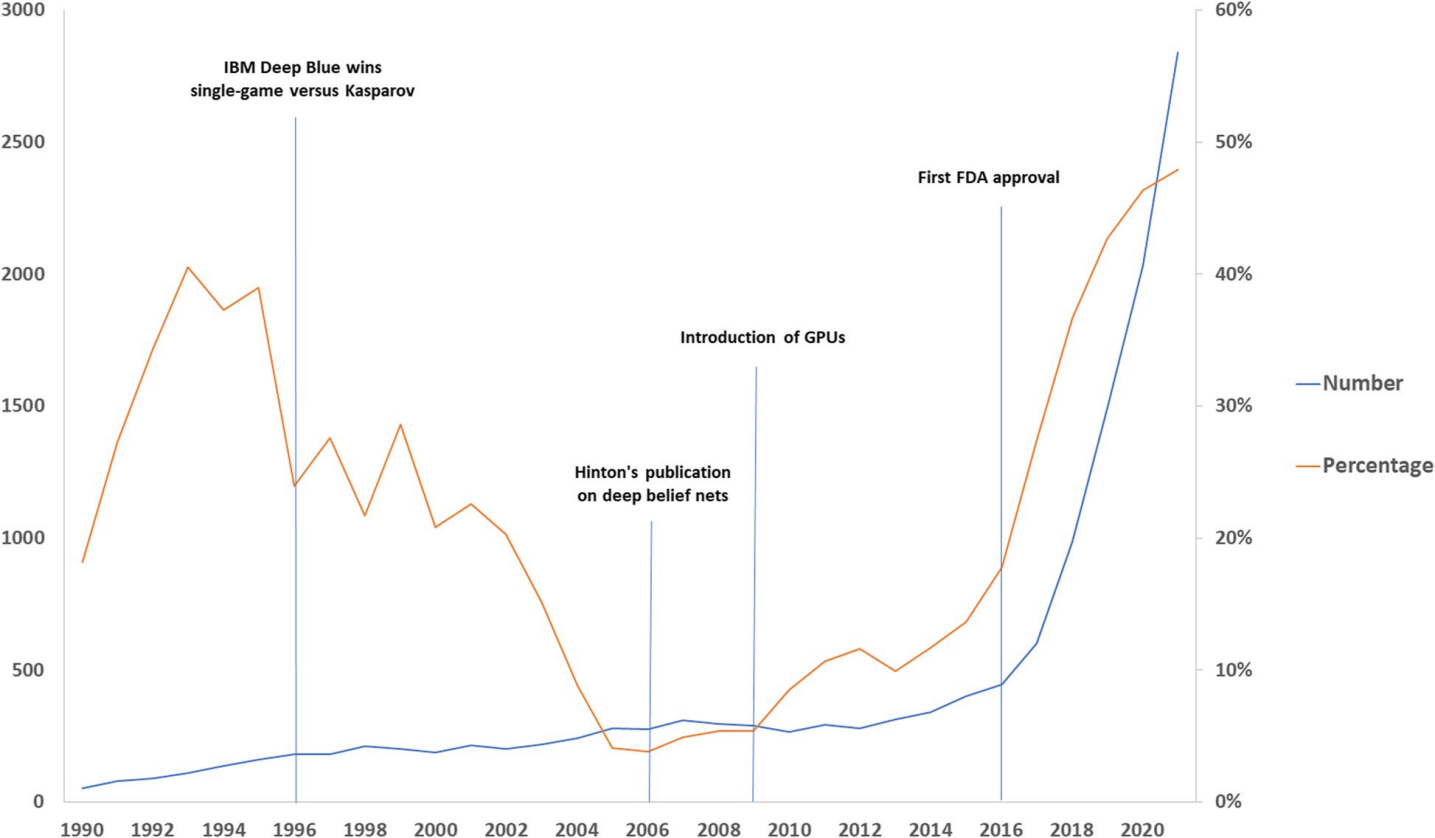
Number of articles on artificial intelligence (AI) in the field of medical imaging and percentage of those declaring the use of AI in the title. Four major milestones are shown. *FDA* Food and Drug Administration, *GPU* Graphics processing unit

Subsequently, we filtered from the retrieved articles those dealing with any diagnostic imaging modality, including radionuclide imaging. To do so, we searched in PubMed using the string (“artificial intelligence” OR “machine learning” OR “deep learning” OR “neural network”) AND (“diagnostic imaging” [MeSH] OR MRI OR CT OR PET OR radiography OR mammography OR ultrasound OR scintigraphy OR DXA OR echocardiography OR angiography OR “radionuclide imaging”). Notably, an even more pronounced U-like-shaped curve appeared (Fig. 3). In particular, the percentage of imaging articles using AI and declaring it in the title went from 41% in 1993 to 4% in 2006, to raise again up to 48% in 2021.

### How to explain the U-like-shaped curve?

The rate of articles using AI and declaring it in the title has decreased from 1990 until 2006 because both rates of neural network and, on much lower values, of AI decreased, with the overall rate being in the middle (Fig. 2). From 2006 until 2014, an almost stationary condition reigned, followed by an increase of both AI and machine learning. Moreover, the deep learning appeared on the scene around 2008 after the Hinton’s publication and pulled up the overall rate [11]. Here we present hypotheses on (1) why we observed the initial decrease and (2) why we observed the latest increase in the rate of AI articles declaring AI in the title.

#### First hypothesis

**Fig. 4:**
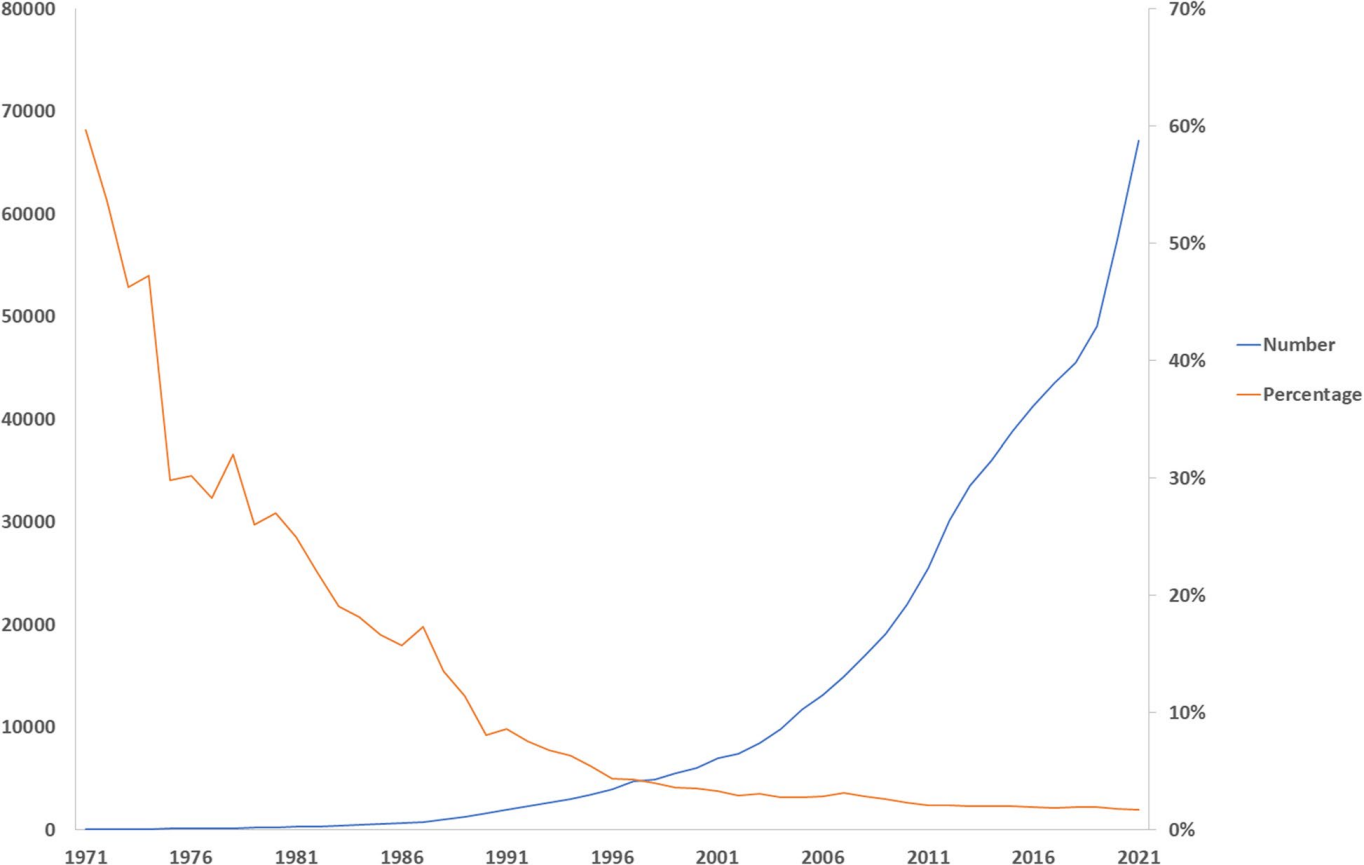
Number of articles using multivariate/multivariable regression analysis and percentage of those declaring this method in the title

We hypothesise that in the first two decades of the observed timeframe, researchers were developing AI techniques itself, *i.e.*, that the AI method development was the main articles’ objective. As such, AI was more frequently declared in the title, although to a lesser and lesser extent while methods were optimised and more established. To support this hypothesis on the establishment of the AI methods, we report the trend of researchers from 1971 to 2021 to declare in the title when they used a statistical approach for prediction, *i.e.*, multivariate/multivariable regression analysis (search string: multivariate OR multivariable; Fig. 4). The absolute number of articles using multivariate or multivariable regression analysis raised progressively but the percentage of those declaring the method itself in the title showed an opposite trend, from about 60% in 1970 to less than 2% in 2021. It is plausible that in the last three decades, multivariate/multivariable regression analysis has been considered an established method [12, 13]. Authors have likely become used to it and gradually renounced to cite it in the title.

#### Second hypothesis

To explain why the percentage of articles declaring AI in the title showed a strong increase from 2014 onwards, we propose that this can be an effect of AI higher and higher performances becoming better than those of humans, stimulating researchers to declare AI in the title, as a sort of “status” labelling their articles. For example, in 2015, AI systems went below the typical 5% error rate of human readers for image recognition [14, 15], especially thanks to the introduction of graphics processing units. Another major milestone was, in 2016, the first Food and Drug Administration approval of a software for analysing magnetic resonance cardiovascular images [16]. The reason for this commitment to declare AI in the title might arise from the need to warn the reader about the complexity of the methods rather than complacency. Especially when an article is published in a medical journal, the readers might not have the needed expertise to fully understand the methods of AI. Also, clinicians tend to use informative titles, that are often recommended, if not required, by the journals themselves. This includes, for example, a declaration of whether an article report on a randomised controlled trial, as mentioned above. In fact, most of the latter declares its nature directly in the title. Moreover, new clinical applications obviously require the inclusion of professionals of medical imaging such as radiologists and nuclear medicine physicians, that likely push for declaring AI in the title, as clearly shown in Fig. 3.

The trend for an increasing percentage of AI declaration in the title might be reaching a peak just in 2020–2021 as shown in Fig. 2. It is difficult to foresee what will happen in the future, whether it will still increase thanks to new applications or to new AI techniques, or if it will decrease as it was for regression analysis.

Our hypotheses could be verified by screening all articles using AI but this would mean reading hundreds of thousands of articles, not an easy task. Maybe an AI system could one day do it easily. Of course, alternative hypotheses can still be true for explaining the data here presented. For instance, the initial decline could be related to a diversification of techniques with more specific terms, such as support vector machines, random forest, or psychological factors like disappointments after the initial hype. In addition, the complexity of the phenomenon here described surely deserves more systematic and detailed search and in-depth analysis. Finally, our search was not systematic, as this is a hypothesis article rather than a research article. Thus, we cannot exclude the possibility that a systematic search could bring to different data, with different time trends.

The discussion just raised by the recent book *The age of AI and our human future* by Henry A. Kissinger, Eric Schmidt, and Daniel Huttenlocher [17] highlights the relevance of AI to the large public and to scientists. In the preface, the authors discuss the “AI’s promise of epoch-making transformations” and say: “AI is not an industry, let alone a single product. It is an enabler of many industries and facets of human life: scientific research, education, manufacturing, logistics, transportation, defence, law enforcement, politics, advertising, art, culture, and more. The characteristics of AI – including its capacity to learn, evolve and surprise – will disrupt and transform them all”. Trends in the declaration of AI in the title of biomedical articles are a small piece of this large landscape.

## Conclusions

In the last 30 years, AI research exploded from some hundreds to over 40,000 articles published every year. The rate of AI declaration in the article title describes a U-like-shaped curve: from near 30% in early 1990s to less than 13% in 2005–2014, subsequently increasing again, up to again 30% in 2020–2021. Data seems to support the hypothesis that the initial decline could be due to the establishment of AI methods, while the recent increase could be attributed to the AI capability to outperform humans as well as to the introduction of new clinical applications.

## Data Availability

Data are available on request.

## Abbreviation

AI: Artificial intelligence

## References

[1] Pesapane F, Codari M, Sardanelli F (2018). Artificial intelligence in medical imaging: threat or opportunity? Radiologists again at the forefront of innovation in medicine. Eur Radiol Exp.

[2] Castiglioni I, Rundo L, Codari M, Di Leo G, Salvatore C, Interlenghi M, Gallivanone F, Cozzi A, D'Amico NC, Sardanelli F (2021). AI applications to medical images: from machine learning to deep learning. Phys Med.

[3] Carlucci Aiello L, Dapor M (2004). Intelligenza artificiale: i primi 50 anni.

[4] Samuel AL (1959). Some studies in machine learning using the game of checkers. IBM J Res Dev.

[5] Fast E, Horvitz E (2017) Long-term trends in the public perception of artificial intelligence. Proceed AAAI Conference Artificial Intell 31

[6] Esteva A, Kuprel B, Novoa RA (2017). Dermatologist-level classification of skin cancer with deep neural networks. Nature.

[7] Hosny A, Parmar C, Quackenbush J, Schwartz LH, Aerts HJ (2018). Artificial intelligence in radiology. Nat Rev Cancer.

[8] McKinney SM, Sieniek M, Godbole V (2020). International evaluation of an AI system for breast cancer screening. Nature.

[9] Rajkomar A, Dean J, Kohane I (2019). Machine learning in medicine. N Engl J Med.

[10] Newquist HP (1994). The Brain Makers: Genius, Ego, And Greed In The Quest For Machines That Think.

[11] Hinton GE, Osindero S, Teh YW (2006). A fast learning algorithm for deep belief nets. Neural Comput.

[12] Schervish MJ (1987). A Review of Multivariate Analysis. Statist Sci.

[13] de Leon-Delgado H, Praga-Alejo RJ, Gonzalez-Gonzalez DS, Cantú-Sifuentes M (2018). Multivariate statistical inference in a radial basis function neural network. Exp Syst Appl.

[14] Russakovsky O, Deng J, Su H (2015). ImageNet Large Scale Visual Recognition Challenge. Int J Comput Vis.

[15] Kaiming H, Xiangyu Z, Shaoqing R, Jian S (2016) Deep Residual Learning for Image Recognition (PDF), in 2016 IEEE Conference on Computer Vision and Pattern Recognition (CVPR). arXiv:770–778 1512.03385

[16] Benjamens S, Dhunnoo P, Meskó B (2020). The state of artificial intelligence-based FDA-approved medical devices and algorithms: an online database. npj Digit Med.

[17] Kissinger HA, Schmidt E, Huttenlocher D (2021). The age of AI: and our human future.

